# Proteome profiling of extracellular vesicles-derived from hepatitis B virus-infected hepatocellular carcinoma cell lines identifies PDCD11 as a carrier of viral RNAs

**DOI:** 10.3389/fcell.2025.1643823

**Published:** 2025-12-05

**Authors:** Indrashish Dey, Anusmriti Das, Subhas Das, Soham Saha, Abhijit Chowdhury, Simanti Datta, Soma Banerjee

**Affiliations:** 1 Centre for Liver Research, School of Digestive and Liver Diseases, Institute of Post Graduate Medical Education and Research, Kolkata, West Bengal, India; 2 Human Genetics Unit, Biological Science Division, Indian Statistical Institute, Kolkata, West Bengal, India; 3 Department of Hepatology, School of Digestive and Liver Diseases, Institute of Post Graduate Medical Education and Research, Kolkata, West Bengal, India

**Keywords:** proteome, extracellular vesicles, EVs, hepatitis B virus, HBV

## Abstract

**Introduction:**

Hepatocellular carcinoma (HCC) is the ultimate result of long-term chronic hepatitis B. Molecular interactions among parenchymal, non-parenchymal, and immune cells in the liver tumor microenvironment (TME) influence the progression of the disease by sharing molecules such as protein, nucleic acids (DNA, coding and non-coding RNAs), lipids, and others through extracellular vesicles (EVs). This study has examined the influence of Hepatitis B virus (HBV) on the enrichment of proteins in the HCC cell-derived EVs and *vice versa*.

**Methods:**

EVs were isolated from conditioned media of stable HBV-containing HepG2.2.15 and HepG2-vector cells using kit and the quality was verified by Nano-particle tracking analysis (NTA) and immunoblotting with EV-specific antibody. Proteome analysis was performed in triplicate with the isolated EV-protein content using label-free LC-MS/MS technology and validated using HBV infected Huh7 and HepG2 cells. Various bioinformatics tools, transfection with Full-length (FL) HBV, anti-sense-oligo (ASO) treatment, immunoblotting, qRT-PCR, chromatin-immunoprecipitation (ChIP), cell proliferation, migration and spheroid formation assays were performed as required. Student’s t-test was performed for statistical analysis.

**Results:**

Proteome data analysis showed that HBV triggered accumulation of 3.4 times more proteins in the EVs-derived from HepG2.2.15 (2,293 proteins) compared to HepG2-vector cells (677 proteins). Differential expression (DE) analysis and subsequent validation with proteomics data of HBV-HCC liver tissue samples revealed enrichment of 103 commonly DE proteins in the EVs of HepG2.2.15 cells. These proteins mostly participated in DNA repair, RNA metabolism, and Golgi trafficking pathways, and these proteins were also overexpressed within cells in presence of HBV. Furthermore, protein-protein network and Hub gene analysis identified 10 key proteins that can interact with other proteins in the network. One of these hub proteins, programmed cell death protein 11 (PDCD11), has been identified as a carrier of HBV-RNA/DNA to the EVs. Depletion of PDCD11 limited the accumulation of HBV-RNAs (pre-genomic RNA, HBx, HBc, HBs mRNAs) and intact virions into the EVs. The FL-HBV genome was detected within EV-enriched virus core particles, which have the potential to infect naïve hepatocytes. Next, integrated transcription factor (TF)-mRNA-miRNA network analysis and validation revealed that TFDP1 transcriptionally upregulated PDCD11, along with other hub proteins, while miR-1-3p has been characterized to suppress their expressions. The binding of TFDP1 to the promoters of the hub genes was further confirmed by ChIP followed by qRT-PCR. Finally, depletion of TFDP1 using ASO and restoration of miR-1-3p in Huh7 cells restricted proliferation, migration, epithelial to mesenchymal transition, and stemness traits in HBV-infected HCC cells.

**Conclusion:**

Proteins enriched in the EVs-derived from HCC cells in presence of HBV may be further investigated to identify novel therapeutic targets within the TME of HBV-HCC. Our findings demonstrated the therapeutic potential of TFDP1-ASO and miR-1-3p, which could lead to new approaches in HBV-HCC treatment.

## Introduction

Hepatocellular carcinoma (HCC) is the most common type of primary liver cancers. It is now the third leading cause of cancer-related mortalities worldwide (Globocan’22). Chronic hepatitis B (CHB) is the prime contributor to the disease. It results from decades of repeated cycles of HBV infection and replication within hepatocytes along with host-immune interactions, followed by the death and regeneration of hepatocytes (Russo et al., 2022; Stein and Loomba, 2009). Although antivirals efficiently restrict the viral copy number, they fail to re-establish the immune milieu resulting in silent progression of CHB towards HCC (Cho and Cheong, 2021; Sangro et al., 2021). Late diagnosis, chemoresistance, and metastasis are the primary unresolved concerns for the clinical management of HCC patients. Only 5% of the early-diagnosed HCC patients with small tumors (multiple nodule of <3 cm in size or one nodule of <5 cm in size) are eligible for curative therapy while multi-tyrosine kinase inhibitors are offered to the advanced HCC patients which extend life expectancy but for 3–6 months only (Pons et al., 2005; Isola and Chen, 2017). Recent evidence depicts that the tumor microenvironment (TME) primarily contributes to this refractory nature of HCC (Li et al., 2023). Hence, understanding the disease pathophysiology is crucial to improving the current treatment regimen for advanced HCC patients.

TME consists of tumor cells, stromal cells, infiltrated immune cells, along with various secretory molecules such as extracellular matrix, cytokines, chemokines, and growth factors, which promote immune evasion, metastasis, and chemo-resistance in HCC cells (Li et al., 2023; Sas et al., 2022). In this microenvironment, extracellular vesicles (EVs) loaded with cell-specific nucleic acids (DNA, mRNA and miRNA, lncRNA, Circular RNA, etc.), protein, lipid, and other molecules play a crucial role in cell-to-cell communication within the TME (Barile and Vassalli, 2017; Witwer and Wolfram, 2021). EVs are phospholipid bilayer-enclosed vesicles of size 30–150 nm released by every cell type after fusion of intracellular vesicles to the cell membrane (Van Niel et al., 2018). Thus, these EVs contain surface markers such as CD63, CD81, TSG101, ALIX, and HSP70, along with cell-specific signatures such as Asialoglycoprotein receptor 1 (ASGR1), which recognizes hepatocyte-derived EVs (Ghosh et al., 2020). EV-enriched miRNAs are used as a promising early diagnostic biomarker for various cancers and to relay messages between cells in the TME as well (Ghosh et al., 2020; Wu et al., 2021; Dai et al., 2020; Mashouri et al., 2019; Sandfeld-Paulsen et al., 2016; Tang et al., 2022; Salciccia et al., 2023; Fang et al., 2023). Enrichment of miRNA-21 in the fibroblast through EVs from HCC cells activates quiescent fibroblasts into cancer-associated fibroblasts (CAFs), and promotes secretion of fibrogenic and angiogenic factors such as TGFβ, MMPs, VEGF, etc. (Zhou et al., 2018). Similar studies have reported M2 polarization of macrophages in tumor milieu by accepting miRNA from EVs of cancer cells through activation of NFκβ pathway (Xu et al., 2022). Very few studies have reported EV-mediated transfer of proteins from cancer cells to immune cells, which induces polarization of M1 macrophages towards anti-inflammatory M2 macrophages (Tan et al., 2023). Thus, a comprehensive analysis of HCC-EVs garners great attention to understand the biology of HCC development and its therapy resistance.

Here, we have reported for the first time the complete proteome profile of the EVs-derived from stable HBV producing HepG2.2.15 cell line and compared with HepG2-vector to investigate the impact of HBV on the loading of cargo molecules to the EVs. The data was compared with the proteome data of HBV-HCC liver tissue samples (Jiang et al., 2019) to classify the enrichment of intracellularly overexpressed proteins into the EVs of HepG2.2.15 cells compared to HepG2-vector cells. The data revealed that HBV fosters 3.4 times more accumulation of proteins in the EVs-derived from HepG2.2.15 cells than HepG2-vector cells. Hub gene analysis with EV-enriched proteins and subsequent analysis revealed that one of the HepG2.2.15-derived EV-enriched RNA binding proteins (RBP), program cell death protein 11 (PDCD11), might be interacting with the of HBV pre-genomic RNA (pgRNA) along with HBx, HBc, and HBs mRNAs. Intact HBV virions were also found in the EVs. These EVs have the potential to infect naïve hepatocytes. The transcription factor TFDP1 and miRNA-1-3p were identified as regulators of PDCD11 along with other hub genes. Thus, overexpression of TFDP1 and depletion of miR-1-3p were associated with increased cell proliferation, migration and sphere-like formation along with expression of the epithelial to mesenchymal transition (EMT) and stemness signature genes.

## Materials and methods

### Maintenance of cell lines

Huh7, HepG2-Vector, and HepG2.2.15 cells were maintained in Dulbecco’s modified Eagle’s medium (DMEM, HiMedia, #AL700A) containing 10% heat-inactivated FBS (ThermoFisher, #26140079), Pen-strep glutamine (100X, ThermoFisher Scientific, # 10378-016) at 37 °C in a humidified chamber containing 5% CO2. Huh7, HepG2 and HepG2.2.15 cells were gifted by Prof. Saumitra Das, Indian Institute of Science, Bangaluru; Prof. Partha Chakraborty, CSIR-Indian Institute of Chemical Biology, Kolkata and Prof. Shyam Kottili, University of Maryland, USA, respectively. HepG2.2.15 is constitutively HBV-producing cell line derived from HepG2 cells and widely used for the study of HBV infected HCC development (Hu et al., 2019).

### Plasmid information

The pre-miRNA clone of miR-1 in pRNAU6.1RNA/Neo vector (pPre-mir-1) was a kind gift from Prof. Raghunath Chatterjee, Indian Statistical Institute, Kolkata, India.

### Cell culture and transfection

The full-length, linear monomeric HBV DNA of subgenotype-D1 was released from pJET1.2/blunt vector by digestion with 1U of SapI/µg at 37 °C for 12 h (Datta et al., 2018; Khatun et al., 2018), followed by gel purification using QIAquick gel extraction kit (Qiagen, # 28704). Huh7 (2 × 10^5^) cells seeded on 24-well plates were individually transfected with 200 ng of the HBV/D1-monomer using Lipofectamine 2000 (Thermo Fisher Scientific, # 11668019). The culture medium was replaced with fresh DMEM at 6 hours post-transfection. Cells were harvested after 48 h of transfection. 100 ng of pPremir-1 and TFDP1-anti-sense oligo (ASO) were transfected in Huh7 cells seeded in 24well and harvested after 48 h. Each experiment was set in triplicate and repeated twice.

### Isolation of RNA and quantitative RT-PCR

Total RNA was isolated using RNAiso Plus (Takara, #9108), and about 2.5 µg of total RNA was used for cDNA synthesis for mRNA using the Revertaid cDNA synthesis kit [Thermo Fisher Scientific (TFS) #AB1453A] following the manufacturer’s protocols. qRT-PCR was performed with PowerUp™ SYBR™ Green PCR master mix (TFS) in QuantStudio7 (TFS), and analysis was performed as fold change in expression of genes using formula 2^−ΔCt^, where ΔCt = (Ct Gene − Ct Internal control). Sequences of primers are presented in Supplementary Table S1.

### Isolation of EVs

Both HepG2-vector and HepG2.2.15 cells were cultured in 3.5 mL of DMEM with 10% exosome-depleted serum (Gibco, #A27208-03) for 72 h in a T25 flask. Media was collected and centrifuged for 20 min at 5000 RPM to pellet down the cellular debris and other contaminants. The supernatant was filtered with a 0.22 μm filter and subjected to EVs isolation using a kit from ExoCan Healthcare Technologies Pvt. Ltd., following the manufacturer’s protocol. Briefly, solution A (100μL/4 mL media) and solution B (2mL/4 mL media) were added, mixed, and centrifuged at 5,000 rpm for 1 h. EVs pellet was treated with RNaseA, DNase and Proteinase K sequentially to remove DNA, RNA and proteins from outside wall of EVs. EVs pellet (soluble protein free) was washed twice with 1 mL of PBS for 10 min, and the final pellet was suspended in 200 μL of filtered PBS for subsequent analysis.

### Isolation of EV proteins

Isolated EVs were subjected to protein extraction using ExoLyseP (ExoCan Healthcare Technologies Pvt. Ltd, India) following the manufacturer’s protocol. Briefly, 50 μL of lysis buffer (ExoLyseP) and 5 μL of protease inhibitor (25x) were added to resuspend the EV pellet. The mixture was incubated at 95 °C for 10 min and then chilled on ice for 5 min to lyse the EVs. The lysate was centrifuged at 8,000 rpm for 5 min, supernatant was collected and preserved at −80 °C freezer in small aliquots.

### Immunoblot analysis

For immunoblot analysis, isolated proteins from EVs were quantified using Bradford reagent (Sigma, #B6916). About 25 μg of protein was boiled with 5xLaemmli buffer for 5 min before loading on the gel. Proteins were separated on 15% SDS-PAGE and transferred onto PVDF membrane (Amersham Biosciences, #GE10600023). After blocking with 5% skimmed milk, the membranes were incubated with primary anti-CD63 antibody (Novus, #NBP2,42225) and anti-Alix (Santa Cruz, #SC53538) at a 1:1,000 dilution overnight. It was then washed with TBS-T, and incubated with secondary antibody (Cell Signaling Technology #7076) at 1:5,000 dilution for 1 h at room temperature, washed, and developed using chemiluminescence kit (Pierce, #32106).

### Nanoparticle tracking analysis (NTA)

To determine the size and concentration of isolated EVs from the cell-cultured supernatant, freshly prepared EVs were subjected to NTA analysis using Malvern Nano Sight NS300 at the Indian Institute of Liver and Digestive Sciences (IILDS), Kolkata, India.

### Proteome analysis: quantification and digestion of EV-protein

EV-protein was subjected to Liquid chromatography-mass spectrometry (LC-MS/MS) analysis from Sandor Proteomics Private Limited, Hyderabad. In brief, an equal amount of protein (100 μg) from respective EVs was diluted with 100 mM ammonium bicarbonate (NH_4_HCO_3_). The sample was briefly centrifuged, treated with 250 mM DTT, vortexed gently, and incubated at 95 °C for 1 h. Then, Iodoacetamide (250 mM) was added, briefly vortexed, and incubated in the dark at room temperature for 45 min. Trypsin was added and digested at 37 °C in a dry bath overnight. The resulting sample was vacuum dried and dissolved in 50 µL of 0.1% formic acid. After centrifugation at 10,000xg, the supernatant was collected into a separate tube.

### Nano UPLC-MSE acquisition

The nanoscale LC separation of tryptic peptides was performed using an ACQUITY UPLC system (Waters Corp., USA). The separation of all samples was performed on an ACQUITY UPLC BEH C18 column (Waters, USA) (150 mm × 2.1 mm × 1.7 µm), an analytical reversed-phase column (Waters, USA). A 10 µL injection volume was used on a BEH C18 UPLC column for the separation of peptides. The samples were initially transferred to the pre-column using an aqueous 0.1% formic acid with a flow rate of 30 mL/min for 1 min. Mobile phase A consisted of 0.1% formic acid in water, and mobile phase B consisted of 0.1% formic acid in acetonitrile. The peptides were separated using a gradient of 2%–80% mobile phase B for 45 min. The column was re-equilibrated to the initial conditions for 15 min. All samples were analyzed in triplicate.

The tryptic peptides were analyzed using a SYNAPT G2 HDMS™ mass spectrometer (Waters, Manchester, UK) with a hybrid quadrupole/ion mobility/orthogonal acceleration time-of-flight (OA-TOF) geometry.

### Data processing and protein identification

The raw data acquired from the instrument was processed using Protein Lynx Global Server (PLGS) PLGS software 3.0.2, and each peptide sequence in FASTA format was matched against the UniProtKB/Swiss-Prot obliged sequences. The criteria considered for analysis were (i) peptide tolerance of 50 ppm, (ii) fragment tolerance of 100 ppm, and (iii) a minimum number of fragments match for peptides and proteins was 2 and 5, respectively. The minimum number of peptides matches for proteins was two. One missed cleavage site was allowed, and the fixed modification of carbamidomethyl-C and oxidation of M were specified. The identification of the protein was performed with a maximum 5% False discovery rate (FDR) in at least three technical replicate injections. For the identification and quantification of protein level, the observed intensity was normalized with the intensity of the identified peptides of the digested internal standard. Proteins with ≥ log_2_fold change ±1 were considered, and protein tables generated by PLGS were merged. The raw file has been deposited to the ProteomeXchange Consortium via the PRIDE partner repository with the dataset identifier PXD056246.

Proteins identified in all three replicates of EVs-derived from HepG2.2.15 vs. HepG2-vector were only counted in this study. The data was validated using various public datasets such as (i) liver tissue proteomics data of HBV-HCC patients, and (ii) Exocarta, a collection of exosomal components (mRNA, lncRNA, miRNA, circRNA, proteins) used to validate enrichment of proteins.

#### Bioinformatics analysis

GO and KEGG pathway analyses were conducted to identify differentially expressed proteins at the biologically active pathways. “DAVID” was used to integrate functional genomic annotations

#### Construction of PPI (protein-protein interaction) and analysis of hub genes

PPI network with identified proteins was constructed using STRING. Genes scoring ≥0.4 were selected from the STRING database to build a network model visualized by Cytoscape (v3.7.2). The Maximal Clique Centrality (MCC) algorithm was used for identifying hub nodes in a co-expression network. A Cytoscape plugin, and CytoHubba were used to determine each node’s MCC, and Hub genes were selected.

#### *In silico* data analysis

UALCAN database (ualcan.path.uab.edu) and TCGA-LIHC data were used for *in silico* analysis of gene expression and overall survival analysis, respectively. Data with log-rank p < 0.05 was considered statistically significant. “Transcript per million” (TPM) is a metric used in RNA sequencing analysis to quantify the expression level of a specific transcript relative to all other transcripts in a sample. OncoDB, a popular database for oncovirus study, was utilized.

#### *In-silico* RNA-protein interaction

RPI-Seq tool was used to identify probable binding sites of protein in RNA. This tool is based on the curated RNA-protein interactions obtained from PRIDB, a database of RNA-protein structures extracted from PDB, and uses Random Forest (RF) and Support vector machine (SVM) as the classifiers. This tool predicted interaction held positive if the score is > 0.5.

#### *In-silico* protein (viral)–protein (host) interaction

To determine the interactions between HBV-encoded proteins and host proteins, the published literature were thoroughly searched considering PubMed and Scopus. Only proteins with experimental validations such as co-immunoprecipitation, yeast two-hybrid, mass spectrometry, and proximity labeling were analyzed. Duplicates were removed. Selected interactions were cross-validated against protein databases (UniProt) for annotation consistency and functional relevance.

### Core particle isolation and quantification of HBV-DNA

Huh7 cells were transfected with HBV-monomer, and after 72 h, intracellular core particles were isolated to extract HBV-DNA as described earlier (Hu et al., 2019; Datta et al., 2018). HBV-DNA was quantified by real-time PCR and normalized with Renilla Luciferase readings. EV-encapsulated core particle was isolated by using the same method after digestion of EVs with DNase I (Roche) and RNase A (TFS) followed by inactivation.

### Migration-assay

Huh7 cells were transfected with desired plasmid in 24 well plate. After 48h, 1 × 10^4^ cells were added to the upper compartment of the 24-well Boyden chambers (8 µm pore, Corning) and 30% serum was added to the bottom chamber as chemo-attractant. The plate was incubated for 12–24 h at 37 °C and non-migrated cells were removed from the top while migrated cells to the lower surface of the membrane were fixed, stained with crystal violet, and counted under a microscope.

### 3D spheroid formation assay

Following the same transfection protocol in Huh7 cells in 24 well plate, cells (1x10^3^/well) were seeded on ultra-low attachment (6-well) plates in serum-free DMEM/F12 medium supplemented with B27 (1×, TFS), EGF (20 ng/mL, TFS), and bFGF (20 ng/mL, TFS). Cultures were maintained at 37 °C in 5% CO_2_, and spheroid formation was monitored for 7–10 days. Spheroids with a diameter ≥50 μm were imaged under a phase-contrast microscope and counted.

### Selection of common transcription factor (TF)

The promoter sequence of each gene was retrieved from UCSC database and −1,000 base pairs from transcription initiation site (+1) were analyzed using TFBIND web tool for searching of TFs TFDP1 was selected as common TF which with binds to all the seven genes.

### Chromatin immunoprecipitation (ChIP) using anti-TFDP1 antibody

After transfection with desired plasmids in 6-well plate for 48h, Huh7 cells (2.5× 10^7^) were crosslinked with 1% formaldehyde for 10 min at room temperature and quenched with 125 mM glycine. Cells were spun down, added lysis buffer and sonicated to an average DNA fragment size of 200–500 bp. Chromatin was incubated overnight at 4 °C with 4 µg anti-TFDP1 antibody (Abclonal, #A5214) or with species-matched IgG as negative control, followed by captured with Protein A/G Sepharose beads. Beads were washed thrice and reverse-crosslinked at 65 °C. DNA was purified by using phenol/chloroform followed by ethanol precipitation and analyzed by qPCR using primers targeting the promoter/enhancer regions of interest. Data were normalized to input and presented as fold enrichment over IgG.

### Selection of common miRNAs

miRNet 2.0 was used to identify the miRNAs-mRNAs (hub genes) interaction. Subsequently, TCGA-LIHC miRNA expression data were used to verify the downregulation of miRNAs (adjusted p < 0.05, log2FC ≤ −0.5). The interaction network was visualized using Cytoscape, and miRNAs were ranked according to the number of targeted hub genes. Expression and survival analyses were performed using the “survival” and “survminer” R packages.

### Identification of target genes for miRNA

Targetscan tool was used to identify the target genes and its binding to the 3′UTR of the identified miRNA.

### Statistical analysis

Statistical calculations were performed using Microsoft Excel and GraphPad PRISM 8 software (GraphPad Software, La Jolla, CA, USA). All data were expressed as mean with standard deviation. To evaluate the differences, the Student’s t-test was performed. P < 0.05 was considered as statistically significant.

## Results

### Comparative proteome analysis of EVs derived from HepG2.2.15 and HepG2-vector cell lines

**FIGURE 1:**
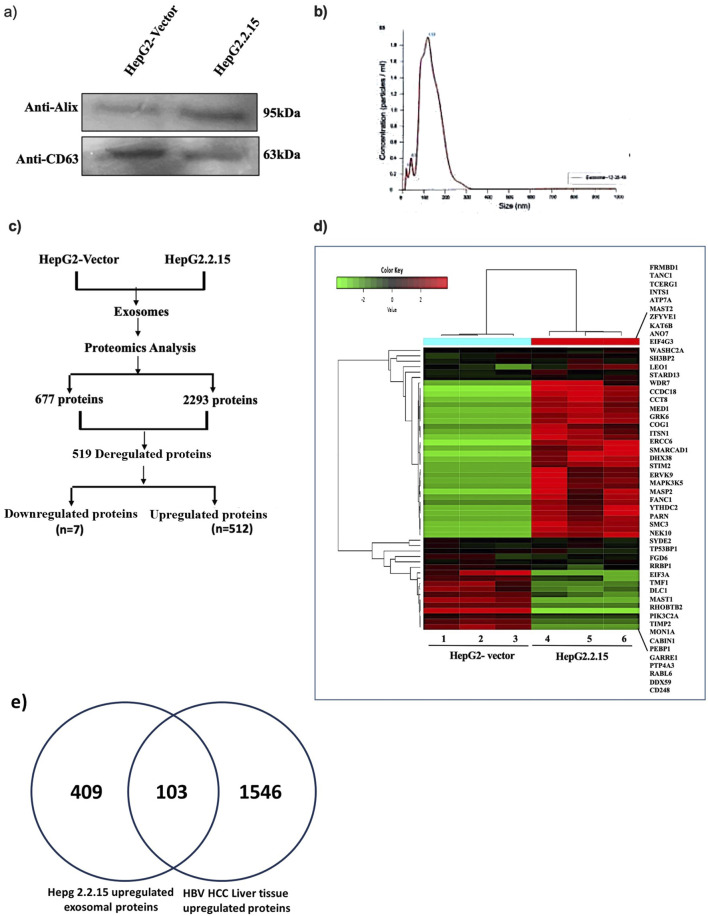
Characterization of EVs and its enriched proteins. EVs isolated from HepG2.2.15 and HepG2-vector were subjected to **(a)** protein extraction and immune blot analysis with anti-CD63, anti-Alix antibody and **(b)** Nanoparticle tracking analysis (NTA) with isolated EVs. **(c)** The flow diagram of proteome analysis performed with exosome enriched proteins, and **(d)** unsupervised hierarchical clustering analysis with top 10% of the proteins displayed in heatmap. Green to red indicates low to high expression, **(e)** Ven-diagram showing DE proteins in HepG2.2.15 *versus* HepG2 and HBV-HCC *versus* control data.

EVs released from HepG2.2.15 and HepG2-vector cells cultured in Exo-free media were isolated using the kit from Exocan Private Limited. The quality and quantity of the EVs were estimated by immuno-blot analysis with anti-CD63, anti-Alix antibodies, and nanoparticle tracking analysis (Figures 1a,b).

To identify the cellular proteins enriched in the EVs of HBV-infected hepatocytes, total protein was isolated from EVs using kit and subjected to proteome analysis (LC-MS/MS) in triplicate. The proteome profiling revealed that a total of 2,293 and 677 proteins were retrieved from the EVs of HepG2.2.15 and HepG2-vector cells, respectively, implicating that the number of proteins released in the presence of HBV infection was 3.4-fold more than in the control cells. Further analysis revealed that 519 proteins were noticed to have differential expression (DE) (|Log_2_FC|>1, p value <0.05) in HepG2.2.15-derived EVs compared to HepG2-vector cells. Among these 519 proteins, 512 proteins were accumulated more in the EVs of HepG2.2.15 cells (>2-fold), and 7 proteins were enriched in the EVs of HepG2-vector cells (>2-fold). Again, 432/512 proteins were explicitly enriched in the EVs of HepG2.2.15 cells, which were absent in the HepG2-vector cells-derived EVs (Figure 1c). The unsupervised hierarchical clustering heatmap represents the top 10% of the proteins in the EVs of HepG2.2.15 cells *versus* HepG2-vector cells (p < 0.05) (Figure 1d).

It is worthy to be noted that we have identified 28 EV-enriched host proteins which might be interacting with various HBV proteins (HBV-polymerase, HBx, HBcore, and HBs) and HBV-cccDNA as identified from published literature (Supplementary Table S2; Supplementary Figure S1).

### Pathway analysis with the EV-enriched proteins

Now, 512 DE proteins in the EVs-derived from HepG2.2.15 were subjected to Gene Ontology (GO) and REACTOME pathway enrichment analysis. GO analysis depicted the biological processes, cellular locations, and molecular functions of the EV-enriched proteins. Interestingly, biological processes revealed that these proteins were mostly contributed to protein depolymerization, RNA splicing, mRNA processing (UPF2, TCERG1, TBL3, PDCD11, CSTF3, DDX23, DHX16, LUC7L3, THOC2, THOC5) and DNA repair (LIG1, POLD1, EXO1, MLH1, MLH3, MSH3, MSH6) (Supplementary Figure S2). Thus, EVs were packaged with nuclear-exported and cytosol-enriched proteins. In-depth molecular function analysis with REACTOME also resonated with this result. In addition to RNA metabolism and DNA mismatch repair pathways, proteins functioning in membrane trafficking and vesicle-mediated transport (TMF1, RALGAPB, MYO5A, COG1, TRIP11, KIF21A, KIF1B, COPG2) pathways were also loaded in the EVs of HepG2.2.15 cells (Supplementary Figure S2).

### Protein-protein interaction (PPI) network construction and selection of hub genes

**FIGURE 2:**
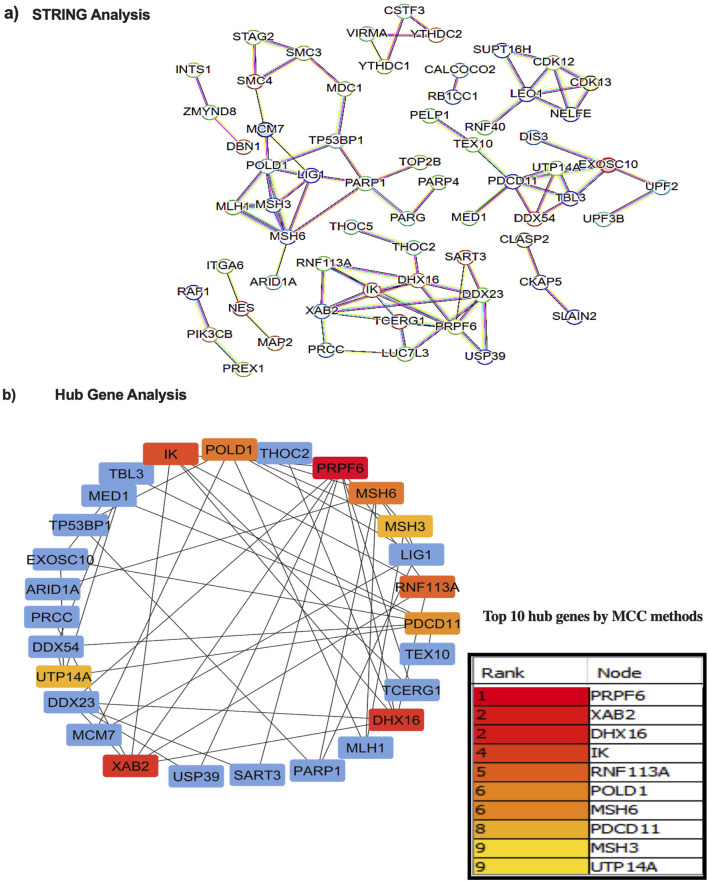
Protein-Protein interaction network and Hub gene analysis. **(a)** STRING database was used to predict interactions among the DE proteins. Nodes represent the proteins in biological networks while edges communicate information about the link between nodes. **(b)** Hub gene analysis to identify the proteins that interact with myriads of proteins and most closely associated with the disease. Top ten hub genes are presented in the table.

Next, the enriched proteins in the EVs of HepG2.2.15 cells were compared with the protein profiles of tumor tissue from HBV-HCC (n = 101) *versus* adjacent tissue (n = 98) to confirm the influence of the DE proteins in the tumor microenvironment (Jiang et al., 2019). The data revealed that 103 EV-enriched proteins were also found to be overexpressed in the liver tissue of HBV-HCC patients (Figure 1e). To gain further insights, high degree hub proteins were determined with these 103 DE proteins using STRING and Cytoscape tools, with a medium confidence score 0.7 for the protein-protein interaction (PPI). The first-order PPI web created an extensive network comprising 63 nodes and 87 edges with an enrichment P value of 1.11 × 10^−16^ (Figure 2a). The node proteins were found to have a significant role in numerous regulatory pathways simultaneously and interact with multiple partners. Next, the maximal clique centrality (MCC) methods were used to select the hub genes from the PPI network using the CytoHubba plugin with default parameters in Cytoscape. The topological network parameters of MCC identified the highest-ranked 10 hub genes, namely, PRPF6, XAB2, DHX16, IK, RNF113A, POLD1, MSH6, PDCD11, MSH3, and UTP14A (Figure 2b).

### Validation and determination of the impact of the hub genes in HCC

**FIGURE 3:**
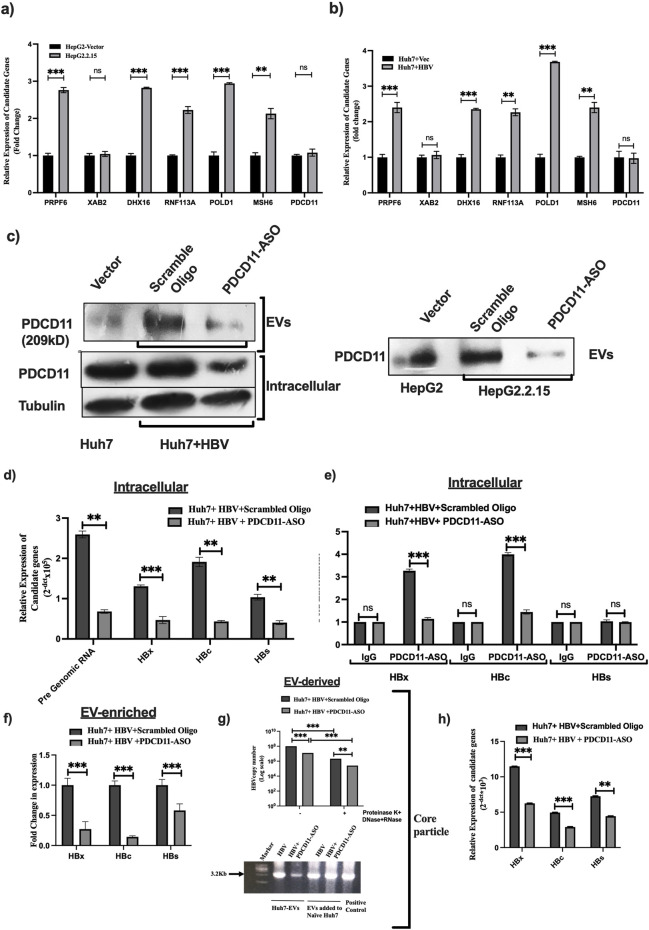
qRT-PCR validation of seven hub genes in **(a)** HepG2-Vector and HepG2.2.15 cells, and **(b)** Huh7+Vector and Huh7+HBV cells. **(c)** Immunoblot analysis with anti-PDCD11 antibody to verify PDCD11 intracellularly and in the EVs upon treatment with PDCD11-ASO in Huh7 cells infected with HBV and HepG2.2.15 cells **(d,f)** Quantification of intracellular and EV enriched pre-genomic RNA, HBx, HBc and HBs mRNAs in HBV infected Huh7 cells treated or kept untreated with PDCD11-ASO. **(e)** RNA-Immunoprecipitation (RIP) analysis with anti-PDCD11 antibody using HBV infected Huh7 cells with or without PDCD11-ASO. **(g)** Amplification of full-length HBV genome (3.2 kb) and quantification of HBV copy number in core particles isolated from HBV-infected Huh7 cell-derived EVs and after incubation of Huh7-derived EVs with naïve Huh7 cells for 48 h. EVs were digested with proteinase K, DNase and RNaseA to remove free RNA/DNA/Protein from outer surface of EVs **(h)** qRT-PCR analysis of HBx, HBc and HBs mRNA from Huh7 naïve cells treated with HBV infected Huh7 cell-derived EVs. p < 0.05 was considered as significant. ** and *** mean *p* < 0.01 and 0.001 while ns depicts not significant.

Next, the expression of 10 hub genes were verified in the TCGA liver cancer dataset. A significant upregulation of all the 10 genes was observed in HCC compared to the adjacent normal tissue. Furthermore, Kaplan-Meier curve analysis and log-rank P values (<0.05) for 10 hub genes depicted that, except for MSH3, the expression level of 9 genes was significantly associated with the overall survival of HCC patients (P < 0.05) (Supplementary Figure S3a). To reinforce the HBV-specific hub gene expression, we reanalyzed the expression of these nine genes in HBV (n = 117) *versus* non-HBV samples (n = 254) of TCGA-LIHC. Four hub genes, namely, PRPF6, RNF113A, POLD1, and MSH6, were found to be significantly upregulated in HBV-infected samples (Supplementary Figure S3b). Thus, we validated the data with qRT-PCR using intracellular total RNA of HepG2.2.15 and HepG2-vector cells as well as Huh7 cells transfected with the monomer of FL-HBV genome compared to Huh7-vector, considering that the EV-enriched proteins were also overexpressed intracellularly. Five genes (PRPF6, DHX16, RNF113A, POLD1 and MSH6) were observed significantly upregulated in presence of HBV (p < 0.05) in both the cell lines while two genes (XAB2 and PDCD11) remained unchanged (Figures 3a,b).

### PDCD11 might serve as a carrier of HBV-specific RNAs to the EVs, and EV-enriched intact virion has the potential to infect neighboring cells

Among all the hub genes, the EV-enrichment of an apoptosis-linked nuclear RNA binding protein (RBP), PDCD11, was further explored. First, we measured the intracellular and EV-enriched PDCD11 protein level in Huh7 and Huh7 transfected with HBV-monomer after treatment with scrambled oligo and PDCD11-ASO. The data was also validated in the EVs-derived from HepG2, HepG2.2.15 cells treated with scrambled oligo and PDCD11-ASO (Figure 3c). The data revealed that PDCD11 remained unchanged intracellularly but was significantly enriched in the EVs upon HBV infection as observed in both Huh7-HBV-monomer *versus* Huh7-vector cells and HepG2.2.15 *versus* HepG2-vector cells while PDCD11-ASO treatment depleted its intracellular and EV-level (Figure 3c). Interestingly, we also quantified intracellular HBV-specific RNAs such as pgRNA, HBx, HBc, and HBs and observed a reduction in their intracellular levels upon PDCD11-ASO challenge compared to control (Figure 3d), suggesting PDCD11 might regulate the expression of those viral RNAs. Thus, RIP assay was performed with anti-PDCD11 antibody using extract from Huh7 cells transfected with HBV-monomer and HBV-monomer + PDCD11-ASO. The qRT-PCR analysis with extracted RNA from RNA-RBP complex revealed that PDCD11-ASO treatment significantly reduced the interactions between PDCD11 and HBx/HBc mRNA, and EVs isolated from the same set of experiments depicted a reduction of HBx/HBc/HBs mRNAs (Figures 3e,f). Next, core particles were isolated from EVs-derived from Huh7 cells transfected with HBV-monomer in presence or absence of PDCD11 after digestion of EVs with RNase, DNase and Proteinase K to remove RNA, DNA and proteins from outside wall of the EVs. The Naïve Huh7 cells were incubated with these EVs for 48h, and isolated core particle-associated HBV-DNA and quantified HBV copy number. Though PDCD11-ASO treatment restricted HBV copy number in Huh7-transfected cells, no significant difference was noticed in naïve Huh7 cells treated with EVs (Figure 3g). This data suggests that EVs of HBV-infected cells might be enriched with infectious HBV-virions, which has the potential to infect naïve Huh7 cells and PDCD11 might be regulating this process. HBV-specific RNAs were also quantified after HBV-infected EV treatment in naïve Huh7 cells and similar results were obtained, i.e., HBx, HBc, HBs specific RNAs were dropped upon reduction of PDCD11 (Figure 3h). The mechanism behind overloading of PDCD11 in the EVs upon HBV infection and its role in persistent HBV infection requires further investigation.

### Transcription factor (TF)-miRNA-mRNA interaction network analysis and validation

**FIGURE 4:**
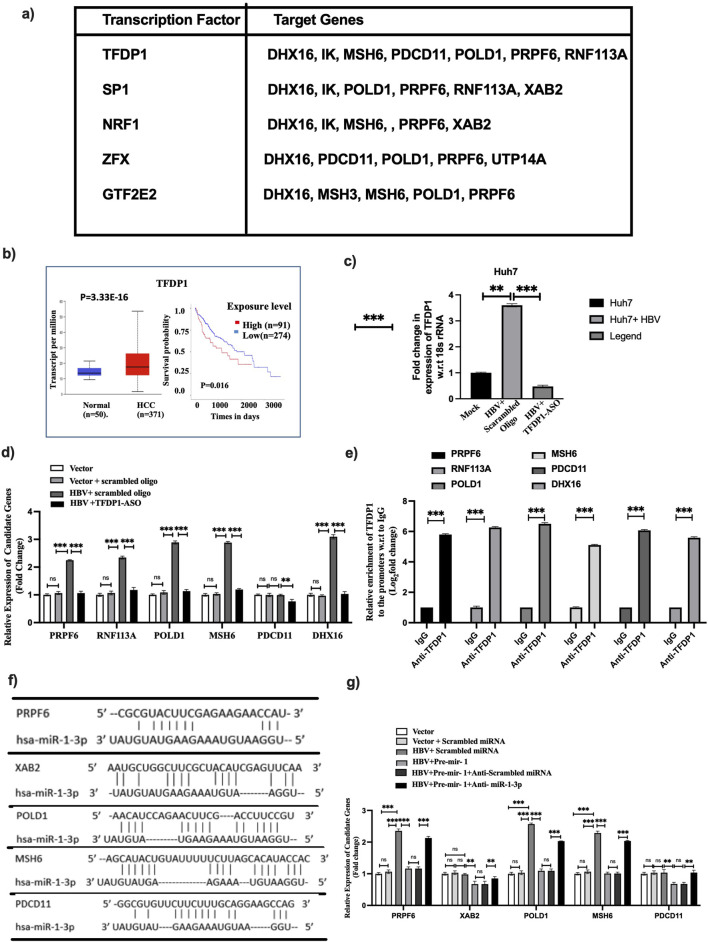
**(a)** List of transcription factors interacting with hub genes. TFDP1 exhibited as highest interacting hub genes. Expression analysis of TFDP1 **(b)** in TCGA-LIHC data with survival analysis, **(c)** in Huh7 cells infected with HBV and Anti-sense Oligo (ASO). **(d)** Impact of TFDP1 on hub genes determined by qRT-PCR upon challenge with PDCD11-ASO in HBV infected Huh7 cells and compared with vector and scrambled oligo treated Huh7 cells. **(e)** ChIP assay with anti-TFDP1 to verify it is binding to the promoters of the hub genes, **(f,g)** Binding of miR-1-3p to the 3′UTR of hub genes identified using Targetscan analysis and verified by qRT-PCR after transfection of pre-miR-1 in Huh7 cells in presence of HBV and compared with anti-miR-1-3p. p < 0.05 was considered as significant. ** and *** mean *p* < 0.01 and 0.001 while ns depicts not significant.

Next, we investigated the transcriptional and post-transcriptional regulation on the expression of PDCD11. As we have identified 10 hub proteins in the EVs-derived from HBV-infected hepatocytes, we aimed to understand the mechanism of enrichment of these ten hub genes in the EVs of the Huh7+HBV and HepG2.2.15. We searched for a common TF for the ten hub genes and the ENCODE ChIP-seq data was analyzed. Five TFs, namely, TFDP1, SP1, NRF1, ZFX, and GTF2E2 were noted to activate the expression of more than 60% of the hub genes, and TFDP1 showed the highest occurrence (Figure 4a). The data was further verified using TFBIND tool and observed that TFDP1 has multiple binding sites on promoter of PDCD11, DHX16, PRPF6, MSH6, POLD1, RNF113A (Supplementary Figure S4). The expression of TFDP1 Figure 4b and other TFs was also validated using TCGA-LIHC data, and observed their higher expression in HCC compared to normal. Survival data also supported the fact that the high expression of TFDP1 was positively associated with poor prognosis of HCC over the period of 5 years (Supplementary Figure S5a). *In vitro* verification of TFDP1 expression disclosed that it was overexpressed in Huh7 cells in presence of HBV and TFDP1-ASO treatment suppressed it (Figure 4c). On the other hand, the expression of six hub genes was decreased upon TFDP1-ASO treatment in Huh7 cells transfected with HBV-monomer compared to scrambled ASO treated cells (Figure 4d). Furthermore, to confirm TFDP1 binding to the promoters of six hub genes such as PRPF6, RNF113A, POLD1, MSH6, PDCD11 and DHX16, ChIP analysis was conducted with anti-TFDP1 antibody and a significant enrichment of TFDP1 was noted in all the six promoters in presence of HBV compared to IgG (Figure 4e).

Next, miRNet2.0 was used to identify the list of miRNAs that could target these hub genes to understand the post-transcriptional regulation. The search criteria were restricted to *Homo sapiens* and only significantly downregulated miRNAs in HCC compared to normal from TCGA-LIHC data were considered. We obtained a total of 20 miRNAs in HCC which were targeting 10 hub genes. Subsequently, a network was constructed with the top 4 miRNAs and 10 hub genes which depicted that hsa-miR-1-3p alone could target 7/9 of the hub genes, while let7b, miR-124-3p, and miR-129-2-3p could target 5, 4, and 3 hub genes, respectively (Table 1; Supplementary Figure S5b). The expressions and survival analyses were performed with TCGA-LIHC datasets for these miRNAs and observed that all the four miRNAs were repressed in HCC compared to adjacent liver tissue. Restoration of each miRNA was associated with significantly improved survival of HCC patients (Supplementary Figure S5c). Here, we have validated the impact of hsa-miR-1-3p on the six hub genes, PRPF6, DHX16, POLD1, PDCD11, MSH6, and XAB2 after restoring its expression in HBV-monomer transfected Huh7 cells. The binding site of miR-1-3p to the 3′UTR of those six genes has been also verified from Targetscan database. A significant downregulation of the target genes was observed in presence of miR-1-3p compared to scramble miRNA and anti-miR-1-3p transfected cells revealed binding of miR-1-3p to the 3′-UTR or coding sequences of the genes (Figures 4f,g). Further functional analysis is needed to confirm the binding of miR-1-3p to the UTRs.

**TABLE 1:** List of miRNAs targeting hub genes with high potential.

miRNA ID	Target genes	Experiment	Tissue
hsa-mir-1-3p	MSH6, POLD1, XAB2, PRPF6, MSH3, PDCD11, UTP14A	Proteomics, Microarrays, RNA-Seq, RPF-seq	Liver
hsa-let-7b-5p	DHX16, PDCD11, XAB2, MSH6, RNF113A, UTP14A	Proteomics, Microarrays, HITS-CLIP, PAR-CLIP	Liver
hsa-mir-124-3p	POLD1, MSH6, PDCD11, UTP14A	Microarrays, HITS-CLIP, RNA-Seq	Liver
hsa-mir-129-2-3p	XAB2, MSH3, MSH6, RNF113A	Microarrays, HITS-CLIP	Liver

**FIGURE 5:**
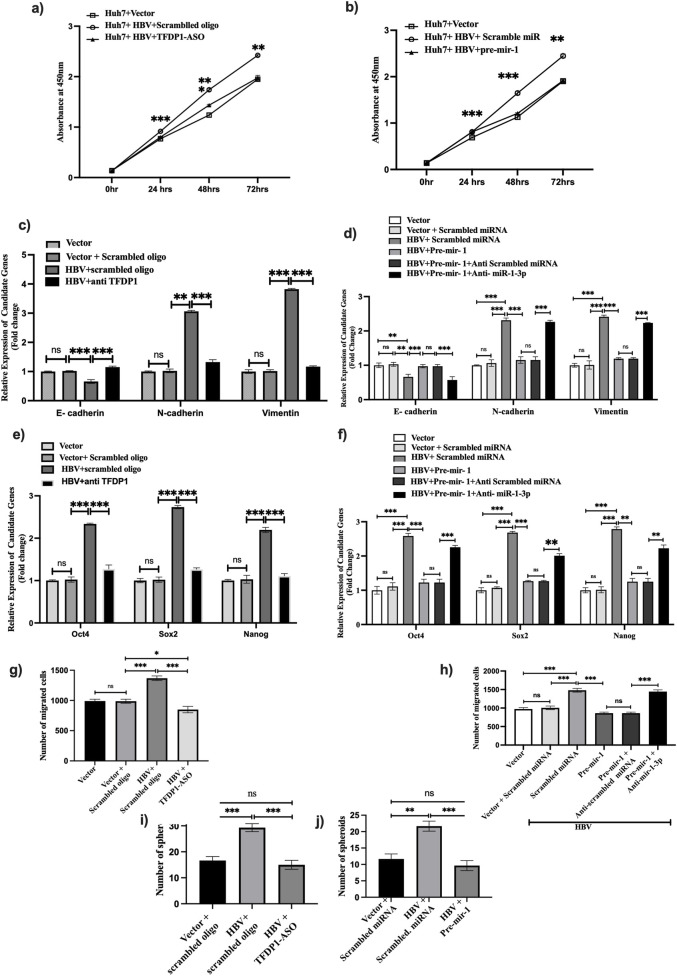
Functional validation TFDP1 and miR-1-3p in Huh7 cells. Cells were transfected with HBV, HBV + Scramble oligo, HBV + TFDP1-ASO and HBV + PremiR-1, HBV + Pre-miR-1+ anti-miR-1-3p and compared **(a,b)** cell proliferation at different intervals, **(c–f)** expression of EMT and stemness markers and **(g–j)** migration and spheroid numbers. **, *** mean *p* < 0.01 and 0.001 respectively. p < 0.05 was taken as significant.

We have also investigated the impact of TFDP1 and miR-1-3p on HCC progression and observed that both depletion of TFDP1 and restoration of miR-1-3p suppressed cell proliferation and reduced expression of EMT (N-Cadherin, Vimentin) and stemness signatures (Oct4, Sox2 and Nanog) in HBV-monomer transfected Huh7 cells (Figures 5a–f). In addition, migration of Huh7 cells and number of spheroid formation in anchorage independent growth condition were significantly dropped upon treatment with TFDP1-ASO and premiR-1 while anti-miR-1-3p restored it in Huh7 cells (Figures 5g–j).

Thus, our findings highlight that HBV triggers accumulation of more proteins in the EVs-derived from infected hepatocytes than controls. HBV-DNA, HBV-RNAs and intact virions are also loaded into the EVs, and EVs-derived from HBV infected cells can infect naïve cells. One of the EV-enriched proteins, PDCD11, might serve as a carrier of HBV-RNA/DNA to the EVs. Most of the hub genes identified in the EVs are upregulated intracellularly by a common TF, TFDP1 while miR-1-3p suppresses these hub genes. This highlights the therapeutic potential of anti-TFDP1 and miR-1-3p for improved treatment in HBV-HCC patients (Figure 5).

## Discussion

Despite limitations in isolation and characterization methods of EVs, the utilization of their content as biomarkers for various diseases and as mediator of cell-to-cell communication in TME has been acknowledged widely (Li et al., 2023; Sas et al., 2022). Ample pieces of evidences suggest that the enveloped viruses like HBV interfere with the molecular mechanism of EV generation and exploit the cargos in the EVs (Wang et al., 2020). We are, for the first time, reporting the proteome of HBV-infected hepatocyte-derived EVs and its impact on disease progression. Using label-free proteome analysis of HepG2.2.15 *versus* HepG2-vector cells revealed HBV induced enrichment of various proteins in the EVs, suggesting viral protein-mediated regulation on the externalization of the cellular proteins through EV synthesis and export. Although, existence of infectious HBV virions and intact core particles have been reported in the HBV-infected cell-derived EVs (Kakizaki et al., 2020; Sanada et al., 2016; Wu et al., 2023), we have shown here that an EV-enriched RBP, PDCD11serves as a carrier of HBV RNA/DNA to the EVs. Using various bioinformatics tools followed by validation revealed TFDP1 transcription factor and miR-1-3p serve as regulators of PDCD11. Depletion of TFDP1 and restoration of miR-1-3p compromised HCC cell proliferation, EMT, stemness, migration and spheroid formation highlighting their importance in HCC therapy.

We analyzed the proteome profile of EVs-derived from HepG2.2.15 and HepG2-vector cells to identify DE proteins that influence TME. The overall data suggests that 3.4-fold more proteins were enriched in the EVs-derived from HepG2.2.15 than HepG2-vector cells. Although HepG2.2.15 cells showed very few HBV copies and viral transcripts, we confirmed our findings by comparing them with the proteomics data from HBV-HCC liver tissue samples, revealing 103 common DE proteins. Interestingly, few proteins were also found to be enriched in the EVs, but remained unaltered intracellularly. These proteins were mostly attributed to RNA metabolism, DNA repair, and mRNA splicing pathways. HBV usually hijacks host DNA repair machinery to repair its relaxed circular genome upon entering the hepatocyte to generate replication-competent covalently closed DNA (cccDNA), and thus, viral proteins transcriptionally upregulate the expression of DNA repair proteins (Wei and Ploss, 2021). The coding and non-coding RNAs are usually escorted by the RNA binding proteins (RBPs) into the EVs (Jia et al., 2017; Wang and Zhang, 2023; Statello et al., 2018), thus, a large number of RBPs were loaded into the EVs-derived from HBV-HCC. Thus, to understand the mechanism of uploading of selective proteins to the EVs, hub gene analysis was employed with 103 proteins and identified 10 hub proteins that can interact with 103 proteins. Interestingly, one of the hub proteins, PDCD11, was noted to be associated with HBV-RNA and DNA, as observed in PDCD11-ASO treatment restricted their loading into EVs and RIP with anti-PDCD11 antibody confirmed their interactions. RNA-protein interaction predictor (RPISeq), and RF-SVM classifier further uncovered that PDCD11 has strong potential to bind to the Enhancer-I and II region of the HBV genome with a score of 0.55–0.9 along with HBx, HBc, and HBs mRNA having a similar score range (data not shown). So, PDCD11 possibly serves as a carrier of HBV-RNA/DNA into the EVs, though further study is required. Enrichment of intact virion particles was also noted in the EVs in presence of PDCD11 and these EVs has the potential to infect naïve hepatocytes. This could be one of the pathways of HBV persistence in CHB patients.

To explore the molecular mechanisms underlying the upregulation of PDCD11 and other hub proteins after HBV infection, a TF–mRNA–miRNA network analysis was performed, which identified TFDP1 and miR-1-3p as key regulators with high interaction potential. The miR-1-3p has been widely reported to act as a tumor suppressor by downregulating multiple oncogenes across different cancer types (Dai et al., 2023; Zhang et al., 2019), whereas TFDP1 functions as a transcriptional co-factor that promotes oncogene expression and tumor progression in several malignancies (Sung et al., 2025; Ju et al., 2025). Our findings indicate that the coordinated dysregulation of these two molecules contributes to HBV-driven oncogenic signaling. Therefore, therapeutic strategies aimed at restoring miR-1-3p expression and reducing TFDP1 activity may provide a synergistic effect to inhibit HBV-associated cancer development. Reduced cell proliferation, migration and expression of EMT and stemness markers upon depletion of TFDP1 and overexpression of miR-1-3p suggest their potential as targets in HCC therapy.

Thus, the overall study highlights that the DNA repair, RNA metabolism, and Golgi trafficking proteins are mostly loaded into the EVs derived from HBV-HCC cell lines, and these proteins are either overproduced by the intracellular machinery or proteins are escorted by other proteins into the EVs. The role of PDCD11 in the shuttling of HBV-RNA/DNA into the EVs may be indispensable, suggesting PDCD11 may be a therapeutic target in HBV-HCC. However, the TF-mRNA-miRNA network analysis with the hub genes and the recent trend of targeting TF in cancer therapy (Bushweller, 2019) highlight the importance of identifying common transcriptional and post-transcriptional regulators for the hub genes, such as TFDP1 and miR-1-3p. This suggests their potential as therapeutic target in HBV-HCC, though further functional validations are necessary.

## Data Availability

The mass spectrometry proteomics data have been deposited to the ProteomeXchange Consortium via the PRIDE partner repository with the dataset identifier PXD056246, available at: https://www.ebi.ac.uk/pride/archive/projects/PXD056246.

## Glossary

3D: Three dimensional
ACQUITY UPLC: ACQUITY Ultra Performance Liquid Chromatography
ALIX: ALG 2 interacting protein X (EV marker)
ASGR1: Asialo glycoprotein receptor 1
ASO: Antisense oligonucleotide
AU: Arbitrary unit (if used)
BEH C18: Bridged Ethyl Hybrid C18 (column)
cccDNA: Covalently closed circular DNA
CD63: Cluster of Differentiation 63 (EV marker)
ChIP: Chromatin immunoprecipitation
DE: Differential expression
DHX16: DEAH-Box Helicase 16
DNase I: Deoxyribonuclease I
DTT: Dithiothreitol
EMT: Epithelial to mesenchymal transition
EV/EVs: Extracellular vesicle(s)
FDR: False discovery rate
FL: Full length
GO: Gene Ontology
HBV: Hepatitis B virus
HCC: Hepatocellular carcinoma
KEGG: Kyoto Encyclopedia of Genes and Genomes
**Log**_**2**_ **FC**: Log_2_ fold change
MCC: Maximal Clique Centrality
miRNA: microRNA
mRNA: messenger RNA
MSH3: MutS Homolog 3
MSH6: MutS Homolog 6
NTA: Nanoparticle Tracking Analysis
OA TOF: Orthogonal Acceleration Time of Flight
PDCD11: Programmed Cell Death 11
PLGS: Protein Lynx Global Server
PPI: Protein protein interaction
PRPF6: Pre-mRNA Processing Factor 6
qRT PCR: Quantitative Reverse Transcription Polymerase Chain Reaction
RBP: RNA binding protein
RIP: RNA immunoprecipitation
rpm: Revolutions per minute
RNF113A: Ring Finger Protein 113A
TF: Transcription factor
TME: Tumor/Tumour microenvironment
TPM: Transcripts per million
UPLC: Ultra-Performance Liquid Chromatography
UTR: Untranslated region
UTP14A: UTP14A Small Subunit Processome Component
XAB2: XPA-Binding Protein 2
IK: Cytokine IK.
UTP14A UTP14A: Small Subunit Processome Component
