# Supplementary material for: Proteome profiling of extracellular vesicles-derived from hepatitis B virus-infected hepatocellular carcinoma cell lines identifies PDCD11 as a carrier of viral RNAs
Source: Front Cell Dev Biol. 2025 Dec 5;13:1643823. doi: 10.3389/fcell.2025.1643823 (PMC12715275; doi:10.3389/fcell.2025.1643823)
Supplement: Supplementary file 2 [file DataSheet1.docx]

**Proteome profiling of extracellular vesicles derived from Hepatitis B virus-infected hepatocellular carcinoma cell lines identifies PDCD11 as a carrier of viral RNAs**

Indrashish Dey *et al*

**Page 2: Table S1**

**Page 3: Table S2**

**Page 4-7: Figure S1-S5**

**Supplementary information**

**Table S1**: List of primers used in the study

| **Primer Name** | **5′-3′ sequence** |
| --- | --- |
| PRPF6 F | GTGGAATGACGCCAGGACTGATG |
| PRPF6 R | CATTGATGTCTCCTCCGTGTGTC |
| DHX16 F | CTCGCCGAGAGTACCTGGCTAAG |
| DHX16 R | GGCATGTGGTAGCGATTGGTGGC |
| XAB2 F | GAAGCATCGCTGTGTGACCGACC |
| XAB2 R | GAGAGTGCTGCGTGATGGGCAG |
| RNF113A F | GAAGGCTGCACTGTGGTTCGAC |
| RNF113A R | CTGTGTCCAGCTCATAGACAGC |
| MSH6 F | CAAGTCTCCGGCGCTGAGTGATG |
| MSH6 R | CTCCGTTGAGGTTCTTCGCCTTG |
| PDCD11 F | CAACGGAGGAGTTGTTAGTCTG |
| PDCD11 R | CAGGCTCAGGTGCACAACTCTG |
| PDCD11 Antisense oligo | TGGATCTTTCTTGTACCTCCTCG |
| POLD1 F | GGGAACTCCTAGCCCTGACT |
| POLD1 R | TTCCGCACTGAGGTCTTCAC |
| Pre-Genomic F | CACCTCTGCCTAATCATC |
| Pre-Genomic R | GGAAAGAAGTCAGAAGGCAA |
| HBX F | TGTGCACTTCGCTTCACCTC |
| HBX R | CAGCCTCCTAGTACAAAGAC |
| HBV Core F | CTGCACTCAGGCAAGCAATT |
| HBV Core R | CTGCGAGGCGAGGGAGTTC |
| HBV Surface F | CTCAGGCCATGCAGTGGAA |
| HBV Surface R | AGAGGACAAACGGGCAACA |
| HBV FL genome F | CCGGAAAGCTTGAGCTCTTCTTTTTCACCTCTGCCTAATCA |
| HBV FL genome R | CCGGAAAGCTTGAGCTCTTCAAAAAGTTGCATGGTGCTGG |
| TFDP1 F | CTTCATCATCGTCAACACCAGC |
| TFDP1 R | GAACCTGCCGTCGTGATGAACAC |
| TFDP1 Antisense oligo | CCGTTGGCTTCAATTAGACCGGCATC |
| PDCD11 PROM F | GCTGCAGCGCACCTAAAGGAG |
| PDCD11 PROM R | GAGGTGAGGCGGATAGGTGCGC |
| DHX16 PROM F | GAGCATGGACCCGATCTTC |
| DHX16 PROM R | GCCGAGCGAGTTCAAACCTCGC |
| PRPF6 PROM F | CCACTCTCACAGACAGGTCTCC |
| PRPF6 PROM R | GAAGCAGAGCGCTCAGTCCGC |
| MSH6 PROM F | CAACGTGAAGGTGAACTGCTGAC |
| MSH6 PROM R | GGCTGGCACGCTGGCGGTGAG |
| POLD1 PROM F | CGCGGAGGATGCGCGCGCAAC |
| POLD1 PROM R | CAGAGCCGCGGCGCTCAGAGAC |
| RNF113A PROM F | GTGATCCGCTCGCCTCGGTCTC |
| RNF113A PROM R | GGAGACCGTCAGCCTGCGAAC |
| Anti-miR-1-3p | A*TACATACTTCTTTACATTCCA  Phophorothioate modified |
| Scrambled Oligo | TATTCTCCCGCCTGTTTTTG |
| Scrambled Anti-sense Oligo | A*ACCACTACACTATGGCACC  Phophorothioate modified |

**Table S2:** List of interacting proteins between HepG2.2.15-cell derived-Extracellular vesicles enriched proteins and HBV cccDNA, HBV- polymerase, Epsilon sequence, HBx, HBcore, HBs and

| **Parameters** | **Interacting Host Proteins** |
| --- | --- |
| **HBV ccc DNA** | SMC5, SMC6, PRMT5, NFIA, SP1, AP1, TBP, PROX1, CREB, NFKB1, POU2F1, NRF1, HNF3A, HNF3B, HNF1A, HNF6 |
| **HBV Polymerase & Epsilon** | RBM24, RBM38, ISG20, DDX17, Hsp70, Hsp40, HOP, PTGES3, APOBEC3G, EEF1A2, DDX3, TBK1 |
| **HBx Protein** | TFIIB, TFIIH, RPB5, DDB1, SMC5, SMC6, NSMCE1, NSMCE2, NSMCE3, NSMCE4A, SLF1, CUL4A, HBXIP, TP53, CREB1, CREBBP, EP300, POLR2E, E4F1, HSPD1, HSPA1A, HSPA8, VCP, BCL2, BCL2L1, NFKBIA, SMAD4, CBFB, PRMT1, PSMA7, PSMC1, PRDX1, EXOSC5, BIRC5 |
| **HBc Protein** | HSP90AA1, DNAJB1, RNF144B (NIRF), NXF1, ALYREF (TREX complex), SRSF1, SRSF9, GIPC1, NPM1 (B23), PIN4 (Par14), PIN4 (Par17, mitochondrial isoform), PIN1, ARID2 (BAF200), SRSF10, MCM2, MCM3, MCM4, MCM5, MCM6, MCM7, NEDD4 |
| **HBs** | SLC10A1 (NTCP), HSPG1 (Heparan sulfate proteoglycan; general family), HSPG2, HSPG3, HSPG4, HSPA8 (HSC70), TBK1 |


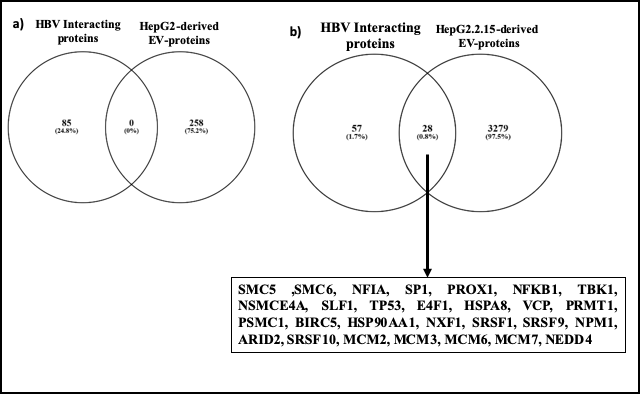


**Figure S1:** Venn diagram between HBV- interacting host proteins and extracellular vesicle enriched proteins in **(a)** HepG2 cells and **(b)** HepG2.2.15 cells.


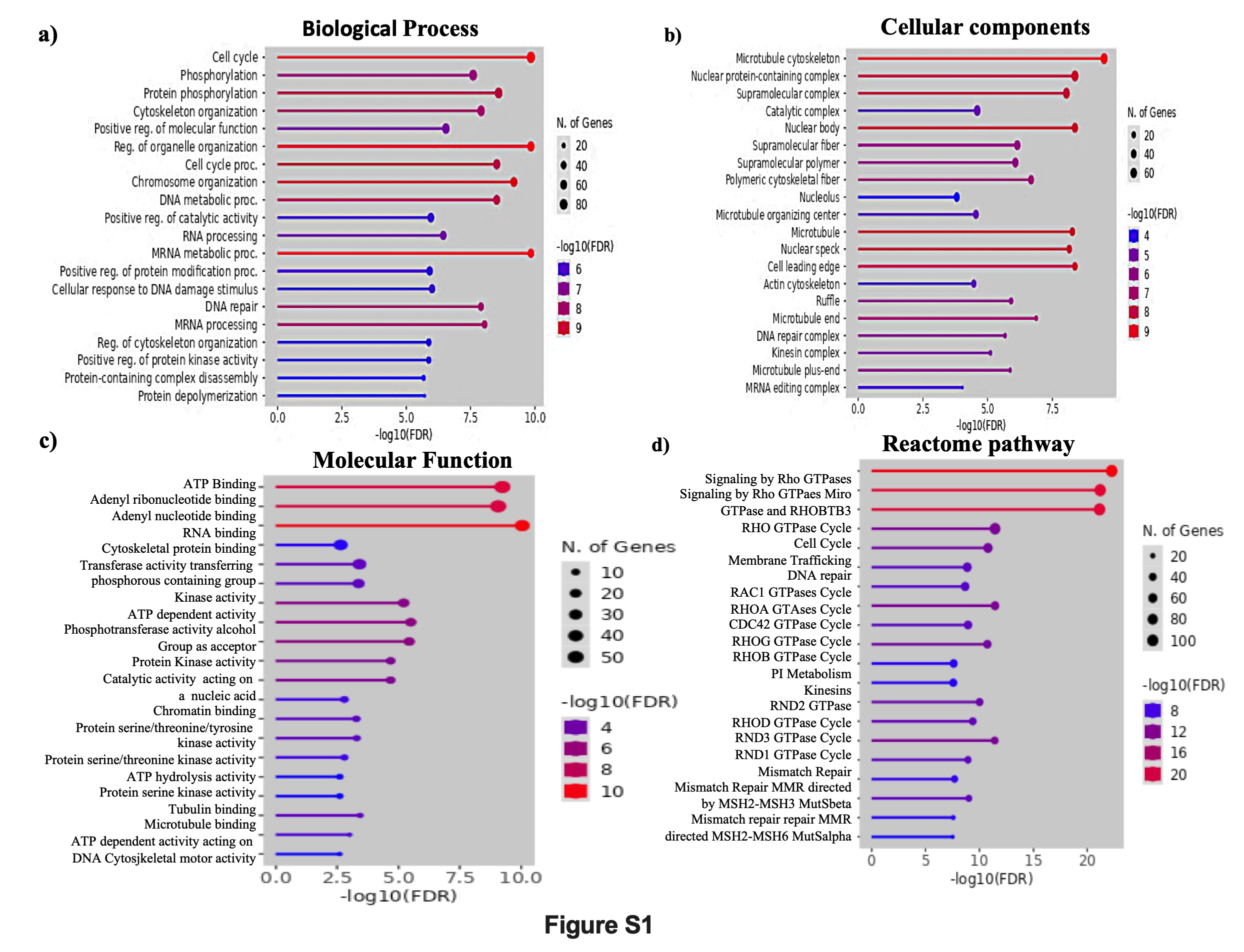


**Figure S2**

**Figure S2:** Identification of pathways and molecular function of differentially expressed (DE) proteins. (**a**) Biological processes, (**b**) Cellular components, (**c**) Molecular function and (**d**) REACTOME pathway.

**Figure S3**

Fi

**Figure S3**: Expression analysis of Hub genes using (**a**)TCGA-LIHC data to determine the expression pattern of Hub genes in HCC vs. normal and also survival curve analysis with high and low expressed Hub genes in HCC, (b) HBV-HCC vs. non-HBV-HCC samples in TCGA-LIHC data. P<0.05 was taken as significant.


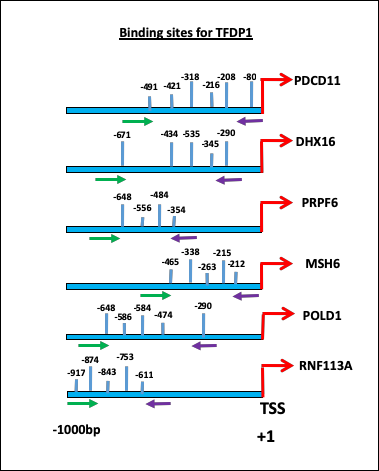


**Figure S4:** Schematic diagram showing the binding site of transcription factor TFDP1 in the promoter of six genes as identified using TFBIND web tool. Red arrow means transcription start site (TSS). Green and violet arrows indicate Forward and Reverse primers used for PCR.

**Figure S5**

**Figure S5**: (**a, c**) Expression and survival curve analysis of transcription factors and miRNAs using TCGA-LIHC data. (**b**) miRNA-mRNA network analysis to identify the best fitted miRNA targeting the hub genes.
